# Central *vs.* Peripheral Cannulation During Reoperations: A Propensity Score Matching Analysis

**DOI:** 10.21470/1678-9741-2022-0463

**Published:** 2023-09-11

**Authors:** Zihni Mert Duman, Ersin Kadiroğulları, Mustafa Can Kaplan, Barış Timur, Aylin Başgöze, Emre Yaşar, Muhammed Bayram, Ünal Aydın, Burak Onan

**Affiliations:** 1 Department of Cardiovascular Surgery, Cizre State Hospital, Sirnak, Turkey; 2 Department of Cardiovascular Surgery, İstanbul Mehmet Akif Ersoy Thoracic and Cardiovascular Surgery Training and Research Hospital, Istanbul, Turkey

**Keywords:** Reoperation, Cardiopulmonary Bypass, Cannulation, Acute Kidney Injury

## Abstract

**Introduction:**

The aim of this study is to compare the postoperative outcomes and early mortality of peripheral and central cannulation techniques in cardiac reoperations using propensity score matching analysis.

**Methods:**

In this retrospective cohort, patients who underwent cardiac reoperations with median resternotomy were analyzed in terms of propensity score matching. Between November 2010 and September 2020, 257 patients underwent cardiac reoperations via central (Group 1) or peripheral (Group 2) cannulation. A 1:1 propensity score matching was performed to balance the influence of potential confounding factors to compare postoperative data and mortality rate.

**Results:**

There were no significant differences when comparing the matched groups regarding early mortality (P=0.51), major cardiac injury (P=0.99), prolonged ventilation (P=0.16), and postoperative stroke (P=0.99). The development of acute renal failure (P=0.02) was statistically less frequent in Group 1.

**Conclusions:**

Performing cardiopulmonary bypass via peripheral cannulation increases acute renal failure in cardiac reoperations. In contrast, peripheral or central cannulation have similar early mortality rate in cardiac reoperations.

## INTRODUCTION

**Table:** 

Abbreviations, Acronyms & Symbols	
CABG	= Coronary artery bypass grafting
CPB	= Cardiopulmonary bypass
ECMO	= Extracorporeal membrane oxygenation
IABP	= Intra-aortic balloon pump
NYHA	= New York Heart Association
PSM	= Propensity score matching
SD	= Standard deviation

Cardiac reoperations have been associated with high mortality and morbidity due to technical difficulties^[1,2]^. Although surgical technique and postoperative care have advanced during the last two decades, the need for cardiac reoperations is still a risk factor for both short-term and long-term mortality^[3]^. The main technical concern during resternotomy is the possibility of iatrogenic fatal injuries to the mediastinal structures below the sternum. In cardiac reoperations, central cannulation is performed after mediastinal adhesion lysis and exposure of cannulation sites. Whereas peripheral cannulation can be done before resternotomy and initiated in case of a major bleeding and hemodynamic instability to decompress the heart^[4]^. So, peripheral cannulation may be a reasonable approach for some surgeons^[4-6]^. Although there are different series in the literature about the outcomes of reoperation regarding coronary or valve procedures^[3,7,8]^, there are still limited data in terms of the outcomes of cannulation strategies and postoperative mortality in cardiac reoperations^[4-6,9]^. The aim of our study is to compare the early mortality and morbidity rates after use of peripheral and central cannulation techniques in cardiac reoperations using a propensity score matching (PSM) analysis.

## METHODS

### Patients

In this retrospective case-control study, patients who underwent cardiac reoperation with median resternotomy in our clinic between November 2010 and September 2020 were evaluated, and 299 patients were identified. Forty-two patients were excluded from the study because they satisfied one or more of the exclusion criteria. The exclusion criteria for this study were as follows: peripheral artery disease (influences the choice of cannulation technique), time between cardiac operations < 30 days, and beating heart surgery. The remaining 257 patients were included in our study. Patients undergoing cardiac reoperation were divided in two groups according to the cannulation strategy. The patients who were operated with the central cannulation technique after resternotomy were determined as Group 1, and patients who were operated with the peripheral cannulation technique before resternotomy were determined as Group 2. The primary endpoint of the study was early mortality. The secondary endpoints of the study were development of acute renal failure, prolonged ventilation, and major cardiac injury. Ethical committee of the hospital approved the study protocol (dated by 11.11.2020, file number 2020/74), and patient consent was obtained.

### Preoperative, Operative, and Postoperative Data

The patients’ files were retrospectively screened and preoperative demographic, clinical, perioperative, and postoperative parameters were evaluated. Diabetes mellitus was defined as a fasting blood glucose > 126 mg/dl in two measurements preoperatively, hemoglobin A1c > 6.5%, or that the patient was being treated with insulin or an oral medication. Obstructive lung disease was determined by a forced expiratory volume in one second, forced vital capacity < 70%, or by the fact that the patient was under bronchodilator treatment. Preoperative renal failure was defined as blood creatinine > 1.5 mg/dL and glomerular filtration rate < 80 ml/min.

Operative times including cardiopulmonary bypass (CPB) and aortic cross-clamping time were reviewed. Postoperative acute renal failure was defined as an increase > 50% in serum creatinine level from the preoperative value or a need for renal replacement therapy. We defined emergency surgery as an operation with a refractory cardiac problem, which will not respond to any treatment other than cardiac surgery, and where there should be no delay in operative intervention. Prolonged ventilation was defined as intubation time > 24 hours. Prolonged inotropic support was determined as the need for one or more inotropic drugs beyond the first 24 hours of operation. Postoperative stroke was defined as brain death, cerebral infarction, intracranial hemorrhage, or seizures. The diagnosis of postoperative cerebral complications was made by computed tomography. Early mortality was defined as death occurring before discharge from the hospital or within 30 days postoperatively.

### Surgical Strategies

The cannulation technique used in each patient was decided at the preoperative daily routine meeting of the surgery team. Defibrillation pads were placed before skin incision. Resternotomies were performed with an oscillating saw. In Group 1, resternotomy was performed first. Pericardial and pleural adhesions were removed with blunt and sharp dissections using electrocautery. Ascending aorta, right atrium, or both cava were cannulated in accordance with the operation plan. In Group 2, the right internal jugular vein was cannulated percutaneously using the Seldinger method. The femoral artery and vein were surgically explored, cannulation sutures were placed and cannulated with the Seldinger method before sternal skin incision. CPB was initiated before sternotomy. Target mean arterial pressure goal was 65 - 75 mmHg during CPB.

After CPB was initiated, isolated or combined cardiac procedures were performed accordingly. Patients were transferred to the intensive care unit and followed-up in a routine surgical care.

### Statistical Analysis

All statistical analyses were performed on R version 4.0.3 (R Foundation for Statistical Computing). Descriptive statistics are reported as percentage for categoric variables and mean standard deviation (SD) for continuous variables. Categorical variables were compared by a chi-squared analysis or Fisher’s exact test. Normal and abnormal continuous variables were compared by Student’s *t*-test and the Mann-Whitney U test. Normally distributed continuous data were presented as mean and SD. Abnormally distributed continuous data were presented as median and interquartile (Q1-Q3). Statistical tests were two-sided, and *P*-values < 0.05 were considered statistically significant.

**Fig. 1:**
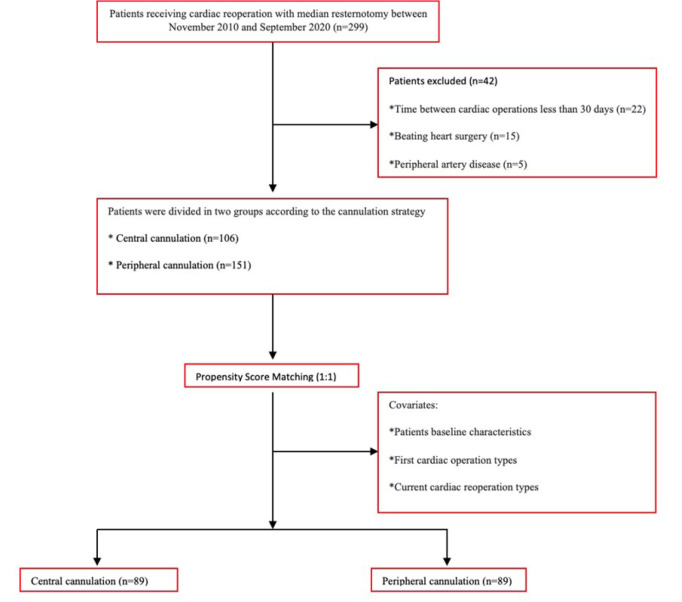
Patient flow diagram used in propensity score matching.

Considering the cannulation technique selection criteria especially for different cardiac operation types cannot be completely random, we applied the PSM method to balance the effect of selection bias and potential confounding factors. PSM analysis was based on the logistic regression model^[10]^. For the purposes of this model, operation types were grouped into four main groups to increase interpretability (“isolated coronary artery bypass grafting [CABG] surgery”, “valve, adult congenital, or intracardiac mass surgery”, “combined CABG and valve surgery”, and “aortic surgery”). The propensity score was calculated according to the patients baseline characteristics (age, sex, ejection fraction, pulmonary artery pressure, New York Heart Association Classes 3 and 4, hypertension, diabetes mellitus, atrial fibrillation, renal failure, obstructive lung disease, infective endocarditis, cerebrovascular disease, emergency operative status, recurrent cardiac operation, and reoperation time < one year), first cardiac operation types ( “isolated CABG surgery”, “valve, adult congenital, or intracardiac mass surgery”, “combined CABG and valve surgery”, and “aortic surgery”), and current cardiac reoperation types (“isolated CABG surgery”, “valve, adult congenital, or intracardiac mass surgery”, “combined CABG and valve surgery”, and “aortic surgery”). Groups were derived using 1:1 matching with a caliper of 0.2. Eventually, a total of 178 patients were matched using PSM analysis. Figure 1 shows that patient flow diagram used in PSM.

## RESULTS

After PSM, the mean age of the patients in Group 1 was 53.0±15.9 years, and in Group 2, it was 52.1±13.9 years (*P*=0.68). There was no statistical difference between the two groups in terms of the baseline characteristics of the patients (Table 1). The rate of patients who had reoperated cardiac reoperation in Group 1 was 13.5%, in Group 2 it was 15.7% (*P*=0.83). In Group 1, nine patients had 2^nd^ time redo sternotomies, and three patients had 3^rd^ time redo sternotomies. In Group 2, six patients had 2^nd^ time redo sternotomies, three patients had 3^rd^ time redo sternotomies, one patient had 4^th^ time redo sternotomies, and one patient had 5^th^ time redo sternotomies.

**Table 1:** Baseline characteristics of the patients in Group 1 (central cannulation) and Group 2 (peripheral cannulation) according to propensity score matching (PSM).

Variable	Before PSM			After PSM		
	Group 1 (N=106)	Group 2 (N=151)	*P*-value	Group1 (N=89)	Group 2 (N=89)	*P*-value
Age (years)	53.1±5.7	53.9±13.1	0.642	53.0±15.9	52.1±13.9	0.68
Sex (female)	60 (56,6)	73 (48.3)	0.192	48 (53.9)	54 (60.7)	0.45
Ejection fraction (%)	53.1±9.9	51.9±9.8	0.373	52.9±9.8	52.9±9.7	0.99
Pulmonary artery pressure (mean, mmHg)	38.3±6.6	39.9±13.3	0.397	39.9±16.7	39.8±13.5	0.93
NYHA Classes 3 and 4	34 (32.1)	62 (41.1)	0.143	28 (31.5)	31 (34.8)	0.75
Hypertension	55 (51.9)	80 (53.0)	0.863	42 (47.2)	43 (48.3)	0.99
Diabetes mellitus	33 (31.1)	40 (26.5)	0.417	24 (27.0)	26 (29.2)	0.87
Atrial fibrillation	31 (29.5)	48 (32.2)	0.648	28 (31.5)	28 (31.5)	0.99
Renal failure	21 (19.8)	32 (21.2)	0.788	19 (21.3)	17 (19.1)	0.85
Obstructive lung disease	17 (16)	39 (25.8)	0.061	14 (15.7)	9 (10.1)	0.37
Infective endocarditis	17 (16)	27 (17.9)	0.699	16 (18.0)	19 (21.3)	0.71
Cerebrovascular disease	9 (8.5)	17 (11.3)	0.469	8 (9.0)	9 (10.1)	0.99
Emergency operative status	14 (13.2)	20 (13.2)	0.993	12 (13.5)	13 (14.6)	0.99
Reoperated cardiac operation	12 (19.8)	22 (14.6)	0.449	12 (13.5)	14 (15.7)	0.83
Reoperation time < one year	21 (19.8)	25 (16.6)	0.503	16 (18.0)	16 (18.0)	0.99

NYHA=New York Heart Association

Data are presented as mean ± standard deviation or the number of patients as percentage (%). *P*-value < 0.05 is considered as significant

Before PSM, there were statistically more patients whose first cardiac operation was aortic surgery in Group 2 (*P*=0.03). There were statistically more patients who underwent isolated CABG in Group 1 (*P*=0.02). In addition, there were statistically more patients who underwent aortic surgery at reoperation in Group 2 (*P*=0.02). We applied the PSM method to balance the effect of cannulation technique selection bias and potential confounding factors for different types of cardiac operations. After PSM, there was no statistically significant difference among the operation types between the two groups. Operation types for all and propensity-matched cohorts (central *vs.* peripheral cannulation) are summarized in Table 2.

**Table 2:** Operation types for all and propensity-matched cohorts (central *vs.* peripheral cannulation).

Variable	Before PSM			After PSM		
	Group 1 (N=106)	Group 2 (N=151)	*P*-value	Group1 (N=89)	Group 2 (N=89)	*P*-value
First operation type						
Isolated CABG	17 (16)	23 (15.2)	0.861	12 (13.5)	12 (13.5)	0.99
Valve, adult congenital, and intracardiac mass surgery	86 (81.1)	109 (72.2)	0.108	74 (83.1)	75 (84.3)	0.99
Combined CABG and valve surgery	2 (1.9)	9 (6)	0.112	2 (2.2)	0 (0.0)	0.50
Aortic surgery	1 (0.9)	10 (6.6)	0.027^*^	1 (1.1)	2 (2.2)	0.99
Reoperation type						
Isolated CABG	10 (9.4)	4 (2.6)	0.018^*^	2 (2.2)	4 (4.5)	0.68
Valve, adult congenital, and intracardiac mass surgery	87 (82.1)	116 (76.8)	0.208	78 (87.6)	74 (83.1)	0.52
Combined CABG and valve surgery	5 (4.7)	7 (4.6)	0.976	5 (5.6)	1 (1.1)	0.21
Aortic surgery	4 (3.8)	24 (15.9)	0.02^*^	4 (4.5)	10 (11.2)	0.16

CABG=coronary artery bypass grafting; PSM=propensity score matching

Data are presented as mean ± standard deviation or the number of patients as percentage (%)

*P*-value < 0.05 is considered as significant

Before and after PSM, statistically significant differences were found between Group 1 and Group 2 in terms of CPB time (*P*=0.02 *vs. P*=0.03, respectively). There was no statistically significant difference between the groups in early mortality in all cohort and propensity-matched cohorts. Early mortality was observed in 24 (13.5%) of 178 patients in propensity-matched cohorts. Before PSM, prolonged ventilation (*P*=0.03) and the development of acute renal failure (*P*=0.04) were statistically less frequent in Group 1. For propensity-matched cohorts, prolonged ventilation (*P*=0.16) was not statistically significant different between the two groups. After PSM, the development of acute renal failure (*P*=0.02) was statistically less frequent in Group 1. Table 3 shows comparison of the perioperative data for propensity-matched cohorts.

**Table 3:** Comparison of the operative and postoperative data for all and propensity-matched cohorts.

Variable	Before PSM			After PSM		
	Group 1 (N=106)	Group 2 (N=151)	*P*-value	Group1 (N=89)	Group 2 (N=89)	*P*-value
Early mortality	10 (9.4%)	27 (17.9%)	0.06	10 (11.2)	14 (15.7)	0.51
Cardiopulmonary bypass time (min)	136.9±52.1	156.5±76.3	0.02^*^	139.1±53.1	160.5±1.4	0.03
Aortic cross-clamping time (min)	86.94±43.49	96.53±53.142	0.14	89.9±44.8	98.3±49.9	0.28
Major cardiac injury	4 (3.8%)	6 (4%)	0.93	4 (4.5)	3 (3.4)	0.99
Prolonged ventilation	33 (31.1%)	67 (44.4%)	0.03^*^	28 (31.5)	38 (42.7)	0.16
Prolonged inotrope use (> 24 hours)	62 (59.6%)	105 (69.5%)	0.10	55 (63.2)	62 (69.7)	0.43
Pulmonary complications	36 (34%)	64 (42.4%)	0.17	30 (33.7)	41 (46.1)	0.13
Acute renal failure	25 (23.6%)	64 (42.4%)	0.04^*^	21 (23.6)	36 (40.4)	0.02
Re-exploration	19 (17.9%)	39 (25.8%)	0.14	16 (18.0)	26 (29.2)	0.11
New-onset atrial fibrillation	13 (12.3%)	16 (10.6%)	0.68	10 (11.2)	10 (11.2)	0.99
Permanent pacemaker	5 (4.7%)	16 (10.6%)	0.09	4 (4.5)	9 (10.1)	0.25
Gastrointestinal complications	7 (6.6%)	9 (6%)	0.83	6 (6.7)	5 (5.6)	0.99
Postoperative stroke	6 (5.7%)	7 (4.7%)	0.72	4 (4.5)	3 (3.4)	0.99
IABP use	3 (2.8%)	4 (2.7%)	0.94	3 (3.4)	2 (2.2)	0.99
ECMO support	2 (1.9%)	7 (4.6%)	0.24	2 (2.2)	3 (3.4)	0.99
Wound complications	11 (10.4%)	19 (12.6%)	0.59	7 (7.9)	11 (12.4)	0.46

ECMO=extracorporeal membrane oxygenation; IABP=intra-aortic balloon pump; PSM=propensity score matching

Data are presented as mean ± standard deviation or the number of patients as percentage (%)

*P*-value < 0.05 is considered as significant

In group 1, four major cardiac injuries occurred. One was in the ascending aorta, two were in the right ventricle, and one was in the right atrium. Two of the injuries occurred during resternotomy, one during pre-pump dissection, and other one during CPB. In Group 2, three major cardiac injuries occurred. One was in the superior vena cava, one was in the right ventricle, and the other was in main pulmonary artery. All injuries occurred during CPB before aortic cross-clamping. There was no statistically significant difference between the groups in terms of major cardiac injury. No complications related to jugular venous cannulation developed in the clinical follow-ups of the peripheral cannulation group. Wound infection was observed in two patients, and seroma developed in three patients in the femoral region.

## DISCUSSION

With the significant improvement of surgical techniques, mortality rates of reoperations are higher than of first operations^[3]^. Injuries during resternotomy are the most common cause of mortality and morbidity^[11]^. Surgeons have been trying to develop some preventive strategies since compensatory recovery methods for undesirable adverse events may not always be successful^[1]^. One of the preventive methods is to set-up peripheral cannulation technique before resternotomy. This strategy can be used as a safe approach in a case of emergency to save the patient’s life. There are different series in the literature about the cannulation strategies in cardiac reoperations^[4-6,9]^. After applying PSM to balance the effect of selection bias and the effect of potential confounding factors, we observed that performing CPB via peripheral cannulation increases acute renal failure in cardiac reoperations. Therefore, prolonged CPB was the main factor that increases postoperative acute renal failure in cardiac reoperations via peripheral cannulation.

Prior studies have identified heterogeneous data on the impact of cannulation techniques on the development of postoperative acute renal failure in cardiac reoperations. Luciani et al.^[4]^ reported that peripheral cannulation reduced postoperative acute renal failure in the postoperative period. In this study, detailed preoperative demographic characteristics that may affect postoperative acute renal failure were not given and it was not clearly discussed why acute renal failure was less common in the peripheral cannulation group. Other studies by Ata et al.^[5]^, Kuralay et al.^[6]^, and Kindzelski et al.^[9]^ found no difference between the central cannulation and the peripheral cannulation technique in terms of postoperative acute renal failure. To the best of our knowledge, this is the first study to demonstrate that performing CPB via peripheral cannulation increases acute renal failure during cardiac reoperations. Previous studies have identified prolonged CPB time as a risk factor for postoperative acute renal failure^[12-14]^. Kumar et al.^[15]^ have examined the relationship between postoperative acute renal failure and CPB time using meta-analysis techniques. They concluded that the mean duration of CPB was 25 minutes longer in patients with postoperative acute renal failure. In our study, in which we applied PSM, the only preoperative and operative difference between the groups was CPB time.

Hamid et al.^[16]^ reported that the in-hospital mortality rate of re-entry injury in cardiac reoperations was 26%. Contrary to the studies in the literature, our study observed that using the peripheral cannulation technique did not have a reducing effect on major cardiac injury^[4,6]^. Most injuries in the central cannulation group occurred during the pre-pump dissection stage. Roselli et al.^[1]^ demonstrated that injuries occurring in the pre-pump dissection cause poor outcome. When cardiac injury occurs in a decompressed heart with the peripheral cannulation technique, early mortality is thought to be less because the repair is easier and faster.

In the whole cohort and propensity-matched cohorts, there was no difference in early mortality between the groups. Like our study, Ata et al.^[5]^, Luciani et al.^[4]^, and Kuralay et al.^[6]^ found no difference between central cannulation technique and peripheral cannulation technique in terms of early mortality. However, Brown et al. found higher mortality in the peripheral cannulation group. But the rate of use of the peripheral cannulation technique in this study is only 5.5%^[17]^.

In this cohort, peripheral cannulation is mostly preferred in patients with aortic surgery first cardiac operation and reoperation. The injury that may occur during resternotomy is difficult to repair, especially if the aortic grafts are dangerously close to the sternum or accompanied by aortic pseudoaneurysm, which suggests that resternotomy under CPB with peripheral cannulation is preferred in these patients. Central cannulation technique was generally preferred in the patient group whose reoperation was to undergo isolated CABG. It may be that surgeons want to avoid complications due to prolongation of CPB time by not using the peripheral cannulation technique, especially in CABG patients who are planned to internal mammary artery harvesting. In the study, PSM was performed to avoid selection bias of choosing different cannulation techniques according to these operation types.

### Limitations

This retrospective study includes data from a single center and from multiple surgeons. The choice of cannulation technique is left to the surgical team. Therefore, the choice of cannulation technique in patients with similar characteristics may have differed according to the clinical experience of the surgeon. Data on intraoperative cardiac injury were obtained from surgical reports. Therefore, small cardiac injuries were probably underreported. The lack of detailed data, such as the distance between the ascending aorta and the sternum, the amount of postoperative drainage, and blood transfusion, is one of the important limitations of the study.

## CONCLUSION

We observed that performing CPB via peripheral cannulation increases acute renal failure in cardiac reoperations. In contrast, peripheral or central cannulation have similar early mortality rate in cardiac reoperations.

## Abbreviations, Acronyms & Symbols

CABG: Coronary artery bypass grafting
CPB: Cardiopulmonary bypass
ECMO: Extracorporeal membrane oxygenation
IABP: Intra-aortic balloon pump
NYHA: New York Heart Association
PSM: Propensity score matching
SD: Standard deviation
